# Supplementary material for: Reconstructing a B-cell clonal lineage. I. Statistical inference of unobserved ancestors
Source: F1000Res. 2013 Apr 3;2:103. [Version 1] doi: 10.12688/f1000research.2-103.v1 (PMC3901458; doi:10.12688/f1000research.2-103.v1)
Supplement: Reconstructing a B-cell clonal lineage: Antigen Receptor Probabilistic Parser (ARPP) software and Clone K/H FASTA files — These files are supplementary to the paper “Reconstructing a B-cell clonal lineage. I. Statistical inference of unobserved ancestors”, which describes the inference of the unmutated ancestor to a set of clonally related antibody genes. The software that implements the methods described is called ARPP UAI, for antigen receptor probabilistic parser: unmutated ancestor inference. setup.exe is the installer for ARPP UAI. Double click the icon, and follow the steps as indicated in the installation wizard. It will install the program and create an icon on your desktop. The program requires a windows operating system to run. ARPP QuickStart is a very brief manual describing how to run the program and interpret its output. CL558(CloneK).fasta and GenbankIgHC2(CloneH).fasta are data input files that can be used to test the installation. These are the datasets that were used to generate the results in the paper. The first of these is a small clone of human kappa sequences, called Clone K in the paper. The latter is a large clone of human heavy chain sequences, called Clone H in the paper. [file f1000research-2-1266-s0000.tgz › ARPP_UA_Inference_setup.zip!/UA Inference setup/ARRP QuickStart.pdf]

ARRP: UA Inference

Thomas B. Kepler
Department of Microbiology
Boston University School of Medicine
tbkepler@bu.edu

Quick Start Guide

UA Inference reads in human variable region immunoglobulin clone sequences and infers their unmutated ancestor, providing additional statistical information as well. When complete, there will be a large number of intermediate files, as well as the primary output files.

Installation

After download, please unzip the zip archive "UA Inference setup.zip". Double-click the *setup.exe* file within the *UA inference setup* folder. A shortcut to UA Inference will be created on your desktop. Double-click that icon to start the program. Ensure that the sample fasta files are placed in a location on the disk where the user has write permission.

The User Interface

Messages to the user are written in the large box on the right side of the interface.

User fields

load sequence data
The data must be in fasta format (http://en.wikipedia.org/wiki/FASTA_format).
Sequences must have names such that they are unique when truncated to 10 characters.
Sequences must be multiply aligned prior to loading and must all have the same length.

Chain
The user must specify whether the immunoglobulin genes are from the Heavy, Kappa, or Lambda locus.

indel freq.
The user specifies the expected frequency of insertions and deletions among all mutations. The default value is 0.02. If the observed frequency is higher than that specified, the analysis may be rerun with a higher value.

init. mu. freq.
The user specifies the expected mutation frequency in the clone being analyzed. This only sets the initial guess, and need not be particularly accurate. In fact, the default value should work most of the time. The processing of the clone entails estimation of the mutation frequency and numerical integration over the mutation frequency.

infer UCA
This button starts the analysis. During the run, a dnaml.exe window will open. Do not interfere with the process running in the window. The amount of time required to complete this stage (tree inference) rises quickly with the number of sequences.

Output
When the inference is completed, the main output files will be written to the same directory in which the input fasta file was located. Therefore*, the user must have write permission* in that directory.

The files of interest are

UCAGrand.fasta
This file contains a single nucleotide sequence. This sequence has, at each position, the nucleotide with the greatest posterior marginal probability at that position.

UCAGrand.txt
This file is best viewed in a spreadsheet application, such as Microsoft Excel. Each column represents a position in the sequence; each row represents a nucleotide state, or the probable error.

The entry in each cell in the rows labeled A,G,T,C,- gives the posterior marginal probability for the indicated nucleotide state at the indicated position. The entry in the row labeled "Error" gives the probable error given that the nucleotide state with the greatest posterior marginal probability is chosen.

The following table shows an excerpt from this file. In this example, positions 0-3 are inferred (CAGG) with negligible error. At position 307, the most likely nucleotide state is G with marginal probability 0.633. The probable error within rounding, therefore, is 1 – 0.633.

Base	0	1	2	3	…	307	308	309
A	0.00E+00	1.00E+00	0.00E+00	0.00E+00	…	3.40E-01	7.52E-03	1.69E-02
G	0.00E+00	0.00E+00	1.00E+00	1.00E+00	…	6.33E-01	7.52E-03	9.60E-01
T	0.00E+00	0.00E+00	0.00E+00	0.00E+00	…	2.08E-02	9.70E-01	1.40E-02
C	1.00E+00	0.00E+00	0.00E+00	0.00E+00	…	6.86E-03	1.45E-02	8.78E-03
-	0.00E+00	0.00E+00	0.00E+00	0.00E+00	…	2.91E-04	3.19E-04	3.72E-04
Error	0.00E+00	0.00E+00	0.00E+00	0.00E+00	…	3.68E-01	2.99E-02	4.01E-02
