# Supplementary material for: Reconstructing a B-cell clonal lineage. I. Statistical inference of unobserved ancestors
Source: F1000Res. 2013 Apr 3;2:103. [Version 1] doi: 10.12688/f1000research.2-103.v1 (PMC3901458; doi:10.12688/f1000research.2-103.v1)
Supplement: Peer review history

# Peer review: Referee response for version 1

**Published:** 2013-04-23

**Authors:** James Crowe Jr

**Affiliations:** 1 Vanderbilt School of Medicine, Vanderbilt University, Nashville, TN, USA

Current high throughput DNA sequencing technologies, including those for amplicons such as PCR products of antibody gene transcripts, allow for the production of millions or billions of nucleotide sequence files. An intriguing finding that has emerged recently is that B cells of apparent clonal families encoding highly related antibody transcripts, representing what appear to be somatic variants, circulate in the peripheral blood and other tissues. After sequence alignment, it seems very intuitive to infer that highly related sequences actually derived in vivo from single B cell clones. However, as the author points out, there is uncertainty in the inferences made by alignment or conventional phylogenetic tools because of the nontemplated regions of recombined antibody genes, and the high frequency of somatically mutated residues in clones from memory B cells. Current computational tools for identification of likely germline gene segments used in the original recombination are reasonable, but currently there are not adequate tools to determine the likelihood as to whether particular recombined and somatically mutated sequences derived biologically from another less mutated sequence in the repertoire from a sample. This is the gap that the author attempts to fill with the tool described. 

This tool likely has significant limitations, but it is important that such tools be developed and tested, with comparison to biological experiments. As sequencing technologies become ever more efficient, it is likely that increased sequencing depth will allow experimentalists to ‘fill in the gaps’ of these types of proposed phylogenies, offering some level of verification of the accuracy of the inferences. Expression and testing of binding of antibodies in intermediate nodes of these phylogenies could be used to experimentally validate the relevance of the inferences. This type of work is already ongoing in several laboratories aimed at rational vaccine and antibody design. 

I am not a mathematician, so I cannot comment as to whether the statistical methods are really appropriate in this work. I can comment however that there are a number of limitations that arise from biological particulars of antibody gene repertoires that likely need to be accounted for in later iterations of this tool. The possible number and diversity of nontemplated junctional nucleotides is theoretically nearly infinite and position independent, but structural constraints limit the length and type of residues encoded in junctions. In fact, canonical structural configurations of the necks of the hypervariable loops (CDRs) likely limit the sequence diversity that can be observed in peripheral blood expressed antibodies after selection. I am not certain if these structural constraints can be used to constrain the inferred phylogenies generated, but that would be very helpful, since somatic variants are unlikely to violate the common structural determinants of the antibody paratopes in antigen-specific repertoires. Antibody genes contain more mutable codons than many other genes encoding proteins, so the likelihood of coding changes may need to be accommodated. Insertions and deletions occur with reasonable frequency in these genes during the process of somatic hypermutation. Some sequences that arise from somatic hypermutation stimulated by a foreign antigen may be eliminated due to autoreactivity or other selective pressures. I also did not see any methods for dealing with sequencing errors, which are vexing in this context. All of these biologic phenomena affecting antibody repertoires make inference of antibody gene phylogenies especially challenging.

Nevertheless, I find it encouraging that new tools like this are being developed that can be tested, evolved and validated in this area. The sequencing technologies present the practical problem of inferring relationships between observed transcripts already, and laboratory experimentalists need practical tools like this for establishing limited sets of candidate genes to synthesize and study. As larger repertoires from more diverse sets of individuals are obtained, the relevance of these tools will become clear. It is especially intriguing to think that, with sufficient sequencing efforts, we may be able to define all possible commonly expressed antibodies and their phylogenies, not just within individuals but across populations.

I have read this submission. I believe that I have an appropriate level of expertise to confirm that it is of an acceptable scientific standard.

# Peer review: Referee response for version 1

**Published:** 2013-04-22

**Authors:** Austin Hughes

**Affiliations:** 1 Department of Biological Sciences, University of South Carolina, Columbia, SC, USA

The method presented in this paper depends on the assumption that reconstructing somatic rearrangement events occurring in the ancestors of B cell clones can be improved by using phylogenetic methods to infer the unmutated ancestor (UA) of mature antibodies. The methods used to reconstruct the UA are standard phylogenetic methods, but the statistical approach taken ignores biological complexities. First, the process of affinity maturation of antibodies is a selective process, not just the accumulation of mutations. Certain mutations, which increase affinity, are selectively favoured, while those that decrease affinity are eliminated. Thus, the author’s assumption of the independence of nucleotide positions in the sequence seems unjustified, as does the use of the simple Jukes-Cantor model. 

In addition, phylogenetic reconstruction in this case (involving short sequences subject to positive selection, resulting in terminal branches that are very long in comparison to internal branches) is likely to be unreliable. The author cites one study suggesting that ancestral sequence reconstruction may not be highly sensitive to tree topology, but the sequences used in that study may not be directly comparable to the present case. It would have been nice to see some quantitative evidence regarding the influence of tree topology on UA reconstruction with these data. 

Furthermore, if tree topology doesn’t matter for the results, why go through the whole elaborate process of phylogenetic tree reconstruction?

In spite of these reservations, the author is to be congratulated for drawing attention to the potential value of using the information in clonally related sequences for inference of ancestral rearrangement.

I have read this submission. I believe that I have an appropriate level of expertise to confirm that it is of an acceptable scientific standard, however I have significant reservations, as outlined above.

# Peer review: Referee response for version 1

**Published:** 2013-04-09

**Authors:** Deborah Dunn-Walters

**Affiliations:** 1 Department of Immunobiology, King's College London School of Medicine, London, UK

This is a really useful tool for immunoglobulin affinity maturation studies – particularly now that technology enables us to generate large clonal families. 

A minor point, in the file downloads information you state “The program requires a Windows operating system to run.” It would be useful to be more specific – 32 or 64 bit?  Which version of Windows?

I have read this submission. I believe that I have an appropriate level of expertise to confirm that it is of an acceptable scientific standard.
